# Supplementary material for: Deciphering Signatures of Mutational Processes Operative in Human Cancer
Source: Cell Rep. 2013 Jan 31;3(1):246–59. doi: 10.1016/j.celrep.2012.12.008 (PMC3588146; doi:10.1016/j.celrep.2012.12.008)
Supplement: Document S1. Article plus Supplemental Information [file mmc1.pdf]

Cell Press
Open ACCESS

Cell Reports
Resource

Deciphering Signatures of Mutational Processes Operative in Human Cancer

Ludmil B. Alexandrov,[1] Serena Nik-Zainal,[1] David C. Wedge,[1] Peter J. Campbell,[1,2,3] and Michael R. Stratton[1,*]
[1]Cancer Genome Project, Wellcome Trust Sanger Institute, Hinxton CB10 1SA, UK
[2]Department of Haematology, Addenbrooke's Hospital, Cambridge CB2 0QQ, UK
[3]Department of Haematology, University of Cambridge, Cambridge CB2 2XY, UK
*Correspondence: mrs@sanger.ac.uk
http://dx.doi.org/10.1016/j.celrep.2012.12.008

SUMMARY

The genome of a cancer cell carries somatic mutations that are the cumulative consequences of the DNA damage and repair processes operative during the cellular lineage between the fertilized egg and the cancer cell. Remarkably, these mutational processes are poorly characterized. Global sequencing initiatives are yielding catalogs of somatic mutations from thousands of cancers, thus providing the unique opportunity to decipher the signatures of mutational processes operative in human cancer. However, until now there have been no theoretical models describing the signatures of mutational processes operative in cancer genomes and no systematic computational approaches are available to decipher these mutational signatures. Here, by modeling mutational processes as a blind source separation problem, we introduce a computational framework that effectively addresses these questions. Our approach provides a basis for characterizing mutational signatures from cancer-derived somatic mutational catalogs, paving the way to insights into the pathogenetic mechanism underlying all cancers.

INTRODUCTION

All cancer genomes carry somatic mutations. A small minority are "drivers" of oncogenesis that confer selective clonal growth advantage. The remainder are "passengers" that have not been positively selected during the evolution of the neoplasm (Stratton et al., 2009). Global sequencing projects are generating catalogs of somatic mutations from tens of thousands of cancers (Hudson et al., 2010). The mutations within these catalogs are the cumulative result of all the somatic mutational mechanisms, including DNA damage and repair processes, that have been operative during the cellular lineage starting from the fertilized egg from which the cancer patient developed to the cancer cell (Stratton, 2011). Because the large majority of mutations in cancer genomes are believed to be passengers, their patterns are largely unmodified by selection (Rubin and Green, 2009). Thus, the mutational catalog from a cancer cell may be treated as a representative archaeological record bearing the combined imprints or signatures of the mutational processes that have been operative.

Several mutational processes are already known to leave characteristic mutational signatures in the mutational catalogs of cancer cells. For example, analyses of mutated cancer genes in tumors of the lung and skin have shown that the classes of mutations found match those induced experimentally by tobacco carcinogens and ultraviolet light respectively, the major known exogenous carcinogenic influences in these cancer types (Hainaut and Pfeifer, 2001; Pfeifer et al., 2002, 2005). Notably, C:G > A:T transversions predominate in smoking-associated lung cancer, whereas C:G > T:A transitions occurring mainly at dipyrimidines and CC:GG > TT:AA double nucleotide substitutions are common in UV light-associated skin cancers. Thus, strong exposures to exogenous mutagens are known to leave their imprints as mutational patterns in cancer genomes.

In principle, other biological processes may influence the patterns of somatic mutations found in human cancers. There may be additional exogenous mutagenic exposures. For example, many widely used chemotherapeutic cancer treatments are mutagens and some have already been shown to leave a distinctive mutational signature in the genomes of cancers recurring after therapy (Hunter et al., 2006). Moreover, there may be exogenous mutagenic exposures instrumental in the primary etiology of cancer that we are currently unaware of. Endogenous sources of mutagens may also contribute to mutations in cancer. For example, intrinsic cellular processes such as energy metabolism and lipid peroxidation are sources for reactive chemicals (e.g., reactive oxygen species) that cause DNA damage (Pluskota-Karwatka, 2008). These endogenous mutagens are known to generate certain subclasses of mutation and, thus, also might shape mutational catalogs within cancer genomes (Ames and Gold, 1991).

The cell employs repair mechanisms that protect the integrity of the genetic code by alleviating and correcting the effects of exogenous and endogenous mutagens (Berwick and Vineis, 2000). For example, the base excision and nucleotide excision repair pathways act on DNA damage respectively caused by cellular metabolites and a wide variety of helix-distorting DNA lesions (Fuss and Cooper, 2006). These repair processes, in turn, influence the mutational signatures left by DNA damaging agents in the final catalog of mutations. This pertains when the repair processes are fully operative, for example the

 ©2013 The Authors
CrossMark

transcriptional strand bias observed in some mutational catalogs that is conferred by the transcription-coupled component of nucleotide excision repair (van Zeeland et al., 2005). It may also apply when they are malfunctioning, for example in the mutational signatures left by defective DNA mismatch repair (Greenman et al., 2007).

In most human cancer types, the mutational signatures imprinted by DNA damage and repair processes have been subject to very limited characterization. In consequence, our understanding of the underlying mutational processes is poor. Previously, we reported an initial outline of a way to extract mutational signatures from the substitutions found in 21 breast cancer genomes (Nik-Zainal et al., 2012a). In this article, we provide a detailed description of our theoretical model that bridges the gap between mutational catalogs derived from cancer genomes and the mutational signatures contained in these catalogs. Further, we provide a systematic computational framework that can be used for accurately deciphering signatures of mutational processes from mutational catalogs of cancer genomes. We extensively evaluate our framework with simulated and real data, demonstrating that it allows incorporation of a wide variety of different mutation types (e.g., substitutions, indels, strand bias, kataegis, etc.). Our framework is freely available (see Experimental Procedures for details) and robust to a large range of different parameters that define its domain of applicability. Importantly, we demonstrate the applicability of the approach to genome and exome sequences and its potential to identify surprising biological insights.

RESULTS

Modeling Mutational Processes Operative in Cancer Genomes

Somatic mutations are conventionally grouped into four classes; base substitutions, small indels, rearrangements, and copy number changes. These can be further subclassified into biologically meaningful subgroups. For example, base substitutions are often classified into six subtypes; C:G > A:T, C:G > G:C, C:G > T:A, T:A > A:T, T:A > C:G, and T:A > G:C. Classification of substitutions may be further refined by including the sequence context of each mutated base, either 5′ or 3′ or both. For example, a C:G > T:A mutation can be characterized as TpCpG > TpTpG (mutated base underlined and presented as the pyrimidine partner of the mutated base pair) generating 96 possible mutation types (6 types of substitution * 4 types of 5′ base * 4 types of 3′ base). This can be further elaborated by considering the transcriptional strand on which a substitution resides. In principle, similar approaches could be taken for the other major classes of mutation (i.e., indels, rearrangements, and copy number changes) and all classes and subclasses of mutation could be incorporated into one analysis.

For the purpose of mathematical modeling, a limited number of features of a mutational signature need to be selected. The choice of features may be influenced by prior biological knowledge and is constrained by statistical considerations and the available data. In this study, a signature of a mutational process is represented as a discrete probability density function with a domain of preselected mutation features. Mathematically, mutation features can be expressed as a finite alphabet Ξ with K letters (each letter corresponds to a mutation feature) and, by definition, a mutational signature P_1 is a lexicographically ordered K-tuple; $P_1 = [p_1^1, p_1^2, \ldots p_1^K]^T$, where p_1^i is the probability of process P_1 to cause the mutation feature corresponding to the i-th letter of the alphabet Ξ, and because p^i are probabilities:

$$\sum_{k=1}^{K} p_1^k = 1 \text{ and } p_1^k \geq 0,\ k = 1 \ldots K. \quad \text{(Equation 1)}$$

Different cancer genomes can be exposed to a particular mutational process at different intensities. For example, a mutational process could cause 1,000 mutations in one cancer genome while causing 20,000 in another. Hence, a mutational process with signature P_1 has an exposure (i.e., number of mutations caused), e_g^1, in a cancer genome g. Note that the subscript of a signature P_1 matches the superscript of the exposure e_g^1 thus denoting that the exposure e_g^1 associates with signature P_1.

The mutational catalog of a cancer genome, defined over an alphabet of mutation types Ξ, can be mathematically expressed as, m_g, a mapping from a genome g and finite alphabet of mutation types Ξ to a specific nonnegative K-tuple. Further, a cancer somatic mutation catalog can be examined as a linear superposition of the signatures and intensities of exposure of mutational processes active at some point in the lineage of cells leading to the cancer cell, plus added noise due to nonsystematic sequencing or analysis errors. Systematic sequencing and analysis errors will be considered as “synthetic mutational processes” with specific profiles present in some (or all) genomes.

An example of three mutational processes with signatures $P_j = [p_j^1, p_j^2, \ldots p_j^6]^T$, where $j = 1 \ldots 3$, composing the mutational catalog of a single cancer genome, $g = 1$, i.e., $m_g = [m_1^1, m_1^2, \ldots m_1^6]^T$, is shown in Figure 1A. Each of the signatures has a specific distribution over the six base substitutions. The first signature has a substantial proportion of C:G > T:A mutations and contributes, in total, 1,000 mutations to the cancer genome. The second process has a high proportion of C:G > A:T mutations while contributing 1,500 mutations. The third process generates substantial numbers of T:A > C:G mutations and contributes 750 mutations (Figure 1A). The mutational catalog of the cancer genome formed by these three processes, however, does not have any notable or specific features and does not obviously resemble any of the mutational signatures that generated it. It contains, in total, 3,315 mutations, 3,250 (~98%) contributed by the three mutational processes and the remaining 65 (~2%) by white noise corresponding to minor processes or experimental errors in generating the mutation catalog of the genome.

Mathematically, we can express mutational signatures as a matrix (Experimental Procedures), and thus the i-th mutation type m_g^i of the catalog of a cancer genome g can be approximately expressed as the sum of the i-th mutation type of all operative processes and their exposures (ignoring the noise term):

$$m_g^i \approx \sum_{n=1}^{N} p_n^i e_g^n. \quad \text{(Equation 2)}$$

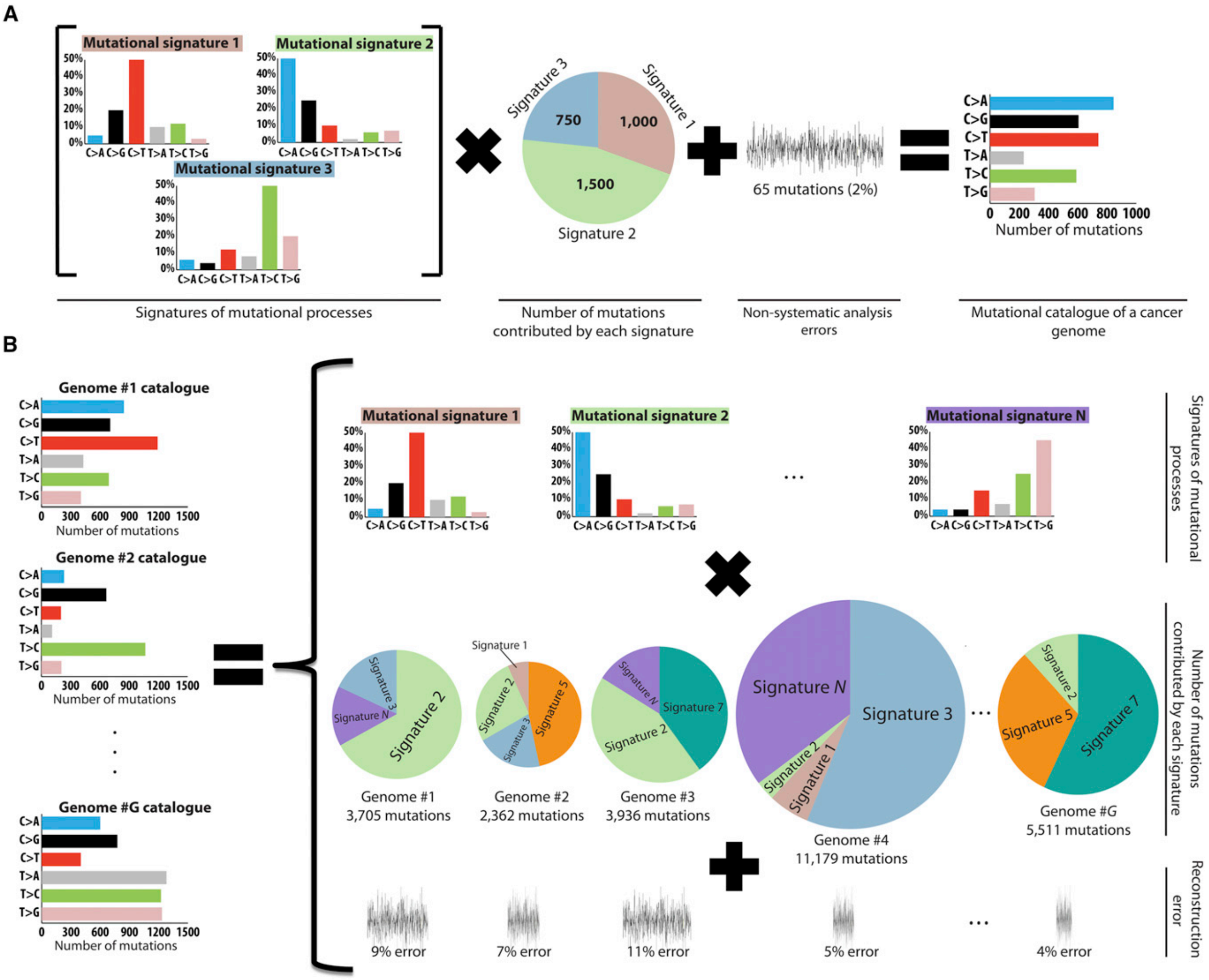

Figure 1. Modeling Signatures of Mutational Processes Operative in Cancer Genomes

(A) Simulated example of three mutational processes operative in a single cancer genome. The mutational catalog of the cancer genome is modeled as a linear superposition of the signatures of the three processes and the respective number of mutations contributed by each signature, plus added nonsystematic noise.

(B) Simulated example illustrating mutational processes operative in a set of G cancer genomes. The mutational catalogs of these G cancer genomes can be used to decipher the signatures of N mutational processes as well as the number of mutations caused by each of the processes in each of the genomes. The extracted signatures and contributions do not allow an exact reconstruction of the original set, thus resulting in genome-specific reconstruction error.

We can generalize Equation 2 for all K mutation types and G genomes by expressing exposures to mutational processes and mutational catalogs as matrices (Experimental Procedures):

$$\begin{bmatrix} m_1^1 & m_2^1 & \cdots & m_{G-1}^1 & m_G^1 \\ \vdots & \vdots & \ddots & \vdots & \vdots \\ m_1^K & m_2^K & \cdots & m_{G-1}^K & m_G^K \end{bmatrix} \approx \begin{bmatrix} p_1^1 & p_2^1 & \cdots & p_{N-1}^1 & p_N^1 \\ \vdots & \vdots & \ddots & \vdots & \vdots \\ p_1^K & p_2^K & \cdots & p_{N-1}^K & p_N^K \end{bmatrix} \times \begin{bmatrix} e_1^1 & e_2^1 & \cdots & e_{G-1}^1 & e_G^1 \\ \vdots & \vdots & \ddots & \vdots & \vdots \\ e_1^N & e_2^N & \cdots & e_{G-1}^N & e_G^N \end{bmatrix}$$

or this equation can be simplified in a matrix form as:

$$M \approx P \times E. \qquad \text{(Equation 3)}$$

Deciphering the Signatures of Mutational Processes from Somatic Mutational Catalogs of Cancer Genomes

The signatures of N different mutational processes and their respective exposures need to be extracted from a set of mutational catalogs M that contain G cancer genomes (Figure 1B). This is equivalent to finding P and E in Equation 3 while only knowing M. The problem can be considered as a specific case of the classic "cocktail party" problem, where multiple people attending a party are speaking simultaneously while several

microphones placed at different locations are recording the conversations. Each microphone captures a mixture of all sounds and the problem is how to decipher the individual conversations from the recordings. This becomes possible because each microphone captures each conversation with a different intensity depending on the distance between the microphone and the conversation. Analogously, provision of a catalog of somatic mutations from a cancer genome provides only the final mixture of the signatures of all mutational processes operative in a cancer sample, and the goal is to decipher these signatures from a set of available mixtures (Figure 1B). Thus, the mutational processes and their signatures are the “conversations,” the exposure to a process is the “loudness of the conversation,” the cancers themselves are the “microphones,” and the final mutational catalogs are the “recordings.”

The cocktail party problem is a type of blind source separation (BSS) problem that involves unscrambling latent (not observed) signals from a set of mixtures of these signals, without knowing anything about the mixing. A number of approaches have been previously developed for solving BSS problems (Comon, 2010) by making specific assumptions about the original sources. The intrinsic nonnegative nature of our BSS cancer genomics problem (see Equation 3) requires a method that assumes (at the very least) nonnegativity of the original sources. One such established approach that has previously been shown to extract biologically meaningful components from complex biological data is nonnegative matrix factorization (NMF) (Lee and Seung, 1999). In this study, we use NMF to solve our BSS cancer genomics problem and decipher signatures of mutational processes from mutational catalogs of cancer genomes.

Extracting Mutational Signatures from Cancer Genomes

An example of applying our theoretical approach to a set of 100 simulated cancer genome mutational catalogs is shown in Figure 2. Similar to many human cancer genomes (Greenman et al., 2007; Nik-Zainal et al., 2012a; Stratton, 2011; Wood et al., 2007), every simulated genome contains between 500 and 50,000 substitutions. The simulated mutations were generated using ten mutational processes with distinct signatures each with 96 mutation types (equivalent to the six substitution types and their immediate 5′ and 3′ sequence context). Poisson noise was added to all simulated data (Experimental Procedures).

Identifying the number, N, of mutational processes operative in a set of cancer genomes is required prior to deciphering their signatures. Our model selection approach identifies N by applying the method for different values of N (Experimental Procedures). For every N, we then evaluate the similarity between the extracted processes (i.e., process reproducibility) from stochastically initialized iterations. Further, for every N, our model selection approach assesses the average Frobenius reconstruction error of the averaged deciphered signatures $\overline{P}$ and their strengths $\overline{E}$, i.e., $\|M - \overline{P}\times\overline{E}\|_F^2$. Low reconstruction error is indicative of an accurate description of the original cancer genome catalogs. We select the value of N for which the extracted processes are reproducible and the reconstruction error is low. Overfitting is avoided by bootstrapping the data (in each iteration) before applying NMF to it (for details see Experimental Procedures).

For the 100 simulated cancer genomes, we are able to identify reproducible solutions for N between two and ten (Figure 2A). Increasing the number of signatures from two to ten substantially reduces the reconstruction error, but increasing beyond ten does not further reduce it (Figure 2A). This indicates that our approach can “optimally” distinguish the signatures of ten mutational processes, precisely the number originally used to simulate the mutational catalogs of the 100 cancer genomes. The ten deciphered signatures are very reproducible (average silhouette width >0.96, Experimental Procedures) as well as extremely similar (average cosine similarity >0.98, see below) to the ones used to generate the 100 mutational catalogs (Figure 2B). Further, our approach was able to accurately identify the number of mutations contributed by each of the ten processes in each of the genomes. Comparison between original and deciphered contributions of one of the signatures in all genomes is shown in Figure 2C whereas a comparison of the contributions of all 10 signatures in a single genome is shown in Figure 2D. A typical comparison between an original and deciphered signature is shown in Figure 2E, whereas a typical comparison between an original and reconstructed mutational catalog of a genome is depicted in Figure 2F.

Identification of Factors that Influence Extraction of Mutational Signatures

To identify factors that affect the ability to extract mutational signatures, we simulated mutational processes under a number of scenarios and compared the deciphered signatures to those used to simulate the data (Experimental Procedures).

To evaluate how the degree of similarity between mutational signatures affects their extraction, we simulated sets of four randomly generated signatures; two were very different from any of the other signatures, whereas the similarity of the remaining two to each other was varied (Figure S1A). A cosine correlation similarity was used as a measure of closeness. This ranges between zero and one, where a similarity of one represents identical signatures and a similarity of zero completely different mutational signatures (Experimental Procedures). Our simulations indicate that 50 or more cancer genomes allow accurate deciphering of signatures that are extremely similar (Figure 3A). Interestingly, however, as few as 20 genomes are adequate to effectively extract signatures that have reasonable similarities between them (Figure 3B).

The number of available genomes mathematically limits the number of signatures that can be extracted. For example, accurately deconvoluting signatures of 15 mutational processes from the mutational catalogs of only ten cancer genomes is ineffective. Simulations with different numbers of genomes and mutational signatures demonstrate that the number of cancer catalogs required for accurately deciphering the signatures operative in them increases exponentially with the number of signatures (Figures 3C and S1B). Thus, although mutational catalogs from 100 cancer genomes are needed to extract the signatures of 15 mutational processes, at least 200 cancer genome catalogs are required for deconvoluting 20 signatures (Figure 3C). Nevertheless, it is possible to decipher at least

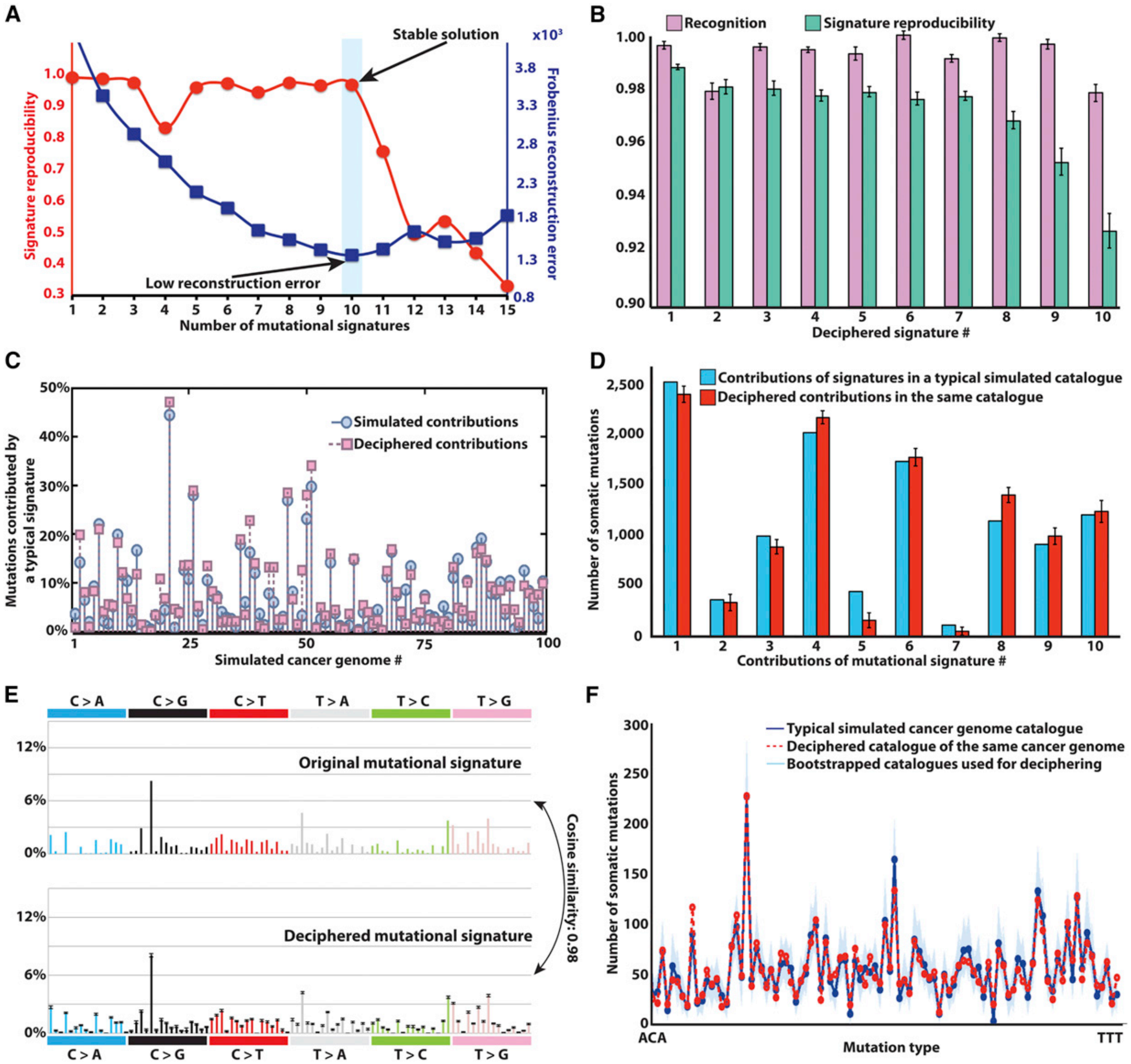

Figure 2. Deciphering Signatures of Mutational Processes from a Set of Simulated Mutational Catalogs from 100 Cancer Genomes

(A) Identifying the number of processes operative in a set of 100 simulated cancer genomes based on reproducibility of their signatures and low error for reconstructing the original catalogs.

(B) Comparison between the ten deciphered signatures and the ten signatures used to simulate the catalogs. Signature recognition, measured using cosine similarity, and signature reproducibility, measured using average silhouette width, is given for each mutational signature. The error bars represent the SD of the corresponding characteristics for the extracted signature(s).

(C) Comparison between deciphered and simulated contributions of one of the ten mutational processes in all cancer genomes.

(D) Comparison between deciphered and simulated contributions of all signatures in a typical cancer genome. The error bars represent the SD of the corresponding characteristics for the extracted signature(s).

(E) Comparison between the profiles of typical deciphered and simulated signature. The error bars represent the SD of the corresponding characteristics for the extracted signature(s).

(F) Comparison between the mutational catalogs of a typical deciphered (red line) and simulated (dark blue line) cancer genome. The separately bootstrapped per iteration mutational catalogs (Experimental Procedures), which are used to decipher the mutational signatures and their contributions, are shown in light blue.

some of the 20 mutational signatures from a set of 100 or fewer mutational catalogs (Figure S1C).

The number of mutations in each cancer catalog affects the ability to decipher signatures of mutational processes. Simulating the mutational catalogs of 50 cancer genomes with different average numbers of mutations indicates that two or three signatures can be effectively extracted from catalogs with very few mutations, whereas extracting seven or more signatures requires an average of at least 1,000 mutations per catalog (Figure 3D). Interestingly, at least 500 mutational catalogs with an average of 96 mutations per catalog (a total of ~50,000 mutations) are needed to decipher five mutational

processes (Figure 3E), but these five mutational processes can be more easily deciphered from 50 cancer genomes containing an average of 480 mutations (a total of ~25,000 mutations, Figure 3D). This result indicates that it is more effective to decipher mutational signatures from a few catalogs containing many mutations than from many catalogs containing few mutations (most likely due to the high relative Poisson variance for small number of mutations).

The strength of exposure of a mutational process in a set of genomes also influences the ability to decipher its signature. Simulations of seven signatures operating with different strengths in 50 mutational catalogs (i.e., exposure to Signature I is fixed whereas the remaining six signatures account for the rest of the mutations) reveal that signatures contributing <5% of all mutations can be difficult to distinguish (Figure 3F). Similarly, deciphering members of a set of mutational signatures that have similar exposures with respect to each other over a set of cancer genomes is also challenging (Figure 3F). To overcome this problem, it may be advantageous to combine sets of mutational catalogs in which mutational processes are more likely to be active in different proportions (e.g., from different cancer types). However, combining sets of mutational catalogs in this way should be considered with caution as the number of cancer genomes required for extraction of signatures increases exponentially with the number of operative signatures and more cancer types may well entail more signatures (Figures 3C and S1B).

In addition to deciphering mutational signatures, our approach extracts the number of mutations contributed by each signature to each cancer genome. Evaluating the average deciphering error for identifying contributions reveals that accurately deciphered mutational signatures (i.e., cosine similarity between simulated and extracted signatures >0.95) are associated with low error for their respective signature contributions (Figures 3F and S1D). Further, the contributions of signatures generating large numbers of mutations (>200) are generally associated with lower error rates (Figure S1E).

Deciphering the Signatures of Mutational Processes Operative in the Genomes of Breast Cancers

We recently described five mutational signatures derived from the 96 possible mutated trinucleotides within the mutational catalogs of 21 whole breast cancer genomes, named Signatures A–E (Nik-Zainal et al., 2012a). Signature A is likely due to deamination of 5-methylcytosine, a relatively well-characterized mutational process. The processes underlying the other signatures are not known, but we have suggested that members of the APOBEC family of DNA/RNA editing enzymes may be responsible for some. Other mutational signatures were detected by visual inspection, including double nucleotide substitutions, a localized base substitution hypermutation phenomenon dubbed kataegis and different patterns of indels occurring either at short tandem repeats or with overlapping microhomologies at breakpoints.

We applied our framework (Experimental Procedures) to the 21 mutational catalogs. This extracted four reproducible mutational Signatures 1–4 (Figure 4A), similar respectively to the previously reported Signatures A, B, D, and E (Nik-Zainal et al., 2012a). However, our new model selection approach and bootstrapping render 21 genomes inadequate to identify the fifth signature with sufficient accuracy. The previously reported mutational Signature C, which is missing from this analysis is quite similar to Signature D, and appears predominantly to have been incorporated here into Signature 3 (Figure 4A). This illustrates the overall reproducibility of the results together with some vulnerability to underlying methodological changes, particularly when the number of genomes is limited and mutational processes are similar to each other.

In principle, our framework can be applied to a wider repertoire of mutation types than the 96 mutated trinucleotides. To demonstrate the potential of this approach, we extended the range of mutation features to include kataegis and double nucleotide substitutions as well as indels at microhomologies and at mono/polynucleotide repeats. Thus, four additional mutational subclasses were incorporated in this model.

Applying this model selection approach revealed five mutational signatures. The substitution patterns of Signatures 1–4 were largely unmodified (Figures 4A and S2). The fifth signature was characterized primarily by kataegis, indicating that kataegis is mostly independent from the other four mutational signatures (Figure 4B). Indels did not have a strong association with Signatures 2 and 5; Signatures 3 and 4 were predominantly associated with indels at microhomologies, whereas Signature 1 associated with nucleotide repeat-based indels (Figure 4C). Double nucleotide substitutions associated mainly with Signature 3 and weakly with the other four signatures. These analyses illustrate the possibility of incorporating additional mutation types and reveal some preliminary associations (and nonassociations) with the previously defined Signatures. However, the numbers of dinucleotides and indels is relatively small and it is therefore unclear if these two mutation classes will keep their current Signature associations or segregate into independent mutational signatures when many more cancer genomes are analyzed.

Our previous analyses showed a transcriptional strand bias for all C:G > A:T mutations across the 21 breast cancer mutational catalogs (Nik-Zainal et al., 2012a). This bias resulted in C > A mutations being more common on the transcribed than the untranscribed strands of genes (and vice versa for G > T). We do not know the cause of this strand bias, but it could be due to past activity of transcription-coupled nucleotide excision repair. We investigated whether a particular mutational signature was associated with the transcriptional strand bias by including information on whether a substitution mutation was on the transcribed or nontranscribed strand, thus increasing the 96 trinucleotide substitutions to 192. Our model selection approach again revealed the signature of four reproducible mutational processes (Figure 5A). The C > A strand bias was not observed in Signatures 2 and 4, but associated with Signature 1 and, to a lesser extent, Signature 3.

Our previous assessment of the impact of sequence context on classification of mutational processes was limited to the bases immediately 5′ and 3′ to each mutated base. However, other sequence motifs close to or distant from the mutant base could be important in defining a mutational process. Here, we have extended the sequence context to include the

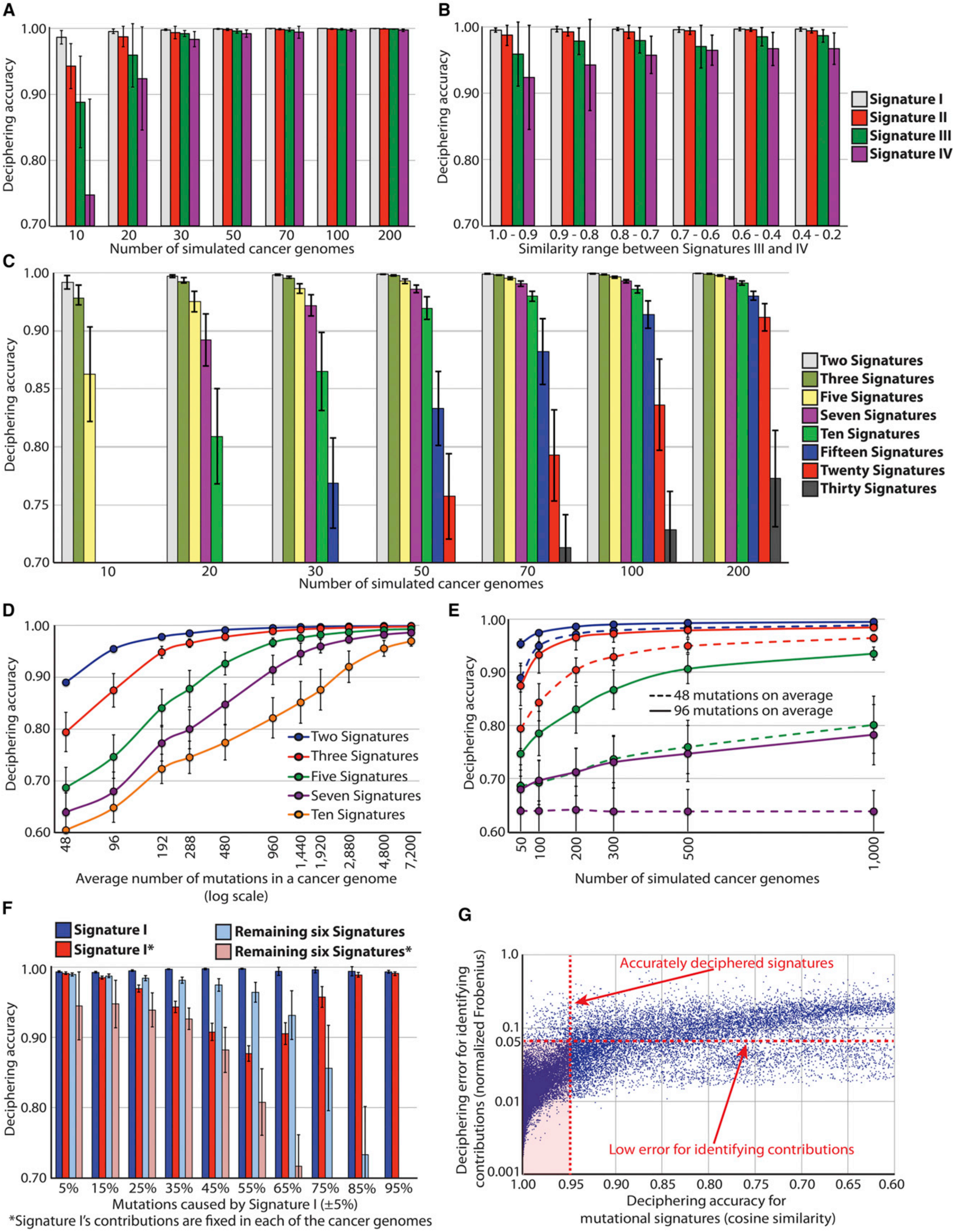

(legend on next page)

two bases 5′ and 3′ to each mutated base, which results in 1,536 possible mutated pentanucleotides. For example, one of the 256 subclasses of C:G > T:A mutation would be …ApTpCpGpC… > …ApTpTpGpC… (mutated base underlined). Our model selection approach is able to find three reproducible mutational processes with these 1,536 mutation types. Analyzing more mutation types leads to fewer mutations per mutation type, thus increasing the relative variability in the bootstrapping procedure (Experimental Procedures), which diminishes the ability of our approach to find the signatures of the operative mutational processes. This limitation should be taken in consideration when choosing the number of mutation types that are being analyzed. Despite this limitation, we can observe new sequence context dependencies in at least one of these processes (Figure 6A). Signature 2 substitutions at TpCpN trinucleotides are dependent on the next base 5′, which is predominantly a pyrimidine (Figures 6A and 6B). Of all C > X at TpCpN mutations caused by Signature 2, 41% are at CpTpCpN, 33% at TpTpCpN, and the remaining 26% are either G or A 5′ to the TpCpN trinucleotide (Figure 6C). Such a tetranucleotide distribution is highly unlikely to happen purely by chance in the human genome (χ^2 test, p value < 0.0001). The result illustrates the richness of detail potentially revealed by this type of analysis, which may be of value in future comparisons of signatures extracted from different cancer types or experimental systems.

Using Mutational Catalogs from Exome Sequencing to Deconvolute Mutational Signatures

The combined protein coding exons (the "exome") constitute ~1% of the human genome. Analysis of exomes compared to whole genome sequences is often perceived as advantageous because of lower cost and because a substantial proportion of cancer-causing driver somatic substitutions, indels, and copy number changes (although not usually rearrangements) may be found using this strategy. As a result, many more exome sequences of cancers are currently being generated than whole genomes.

We therefore assessed the power of our approach to extract mutational processes from exome sequences using 100 recently sequenced breast cancer exomes (Stephens et al., 2012) containing ~7,000 somatic substitutions, ~25-fold fewer than found in the 21 whole cancer genomes. Our framework revealed two reproducible mutational signatures with strong similarities to the previously described Signatures 1 and 2 (Figure 7). Thus, mutational catalogs from exomes can be used to extract mutational signatures, although not with the precision and comprehensiveness provided by the much larger mutation numbers in whole genomes. It is quite possible, however, that increasing the number of exome sequences to a few thousand will allow identification of many mutational processes operative in breast cancer.

Analysis of smaller, exome-derived mutational catalogs (or catalogs from other subcomponents of the genome) may also be useful in detecting biologically revealing features of mutational processes that are particular to coding, transcribed, nontranscribed, or other functionally distinct regions. For example, incorporating transcriptional strand in the analysis of the 100 breast cancer exomes revealed the presence of a context-specific (i.e., TpCpT) strand bias for Signature 2 (Figures 7B and 7C). However, this strand bias is not observed in the version of Signature 2 extracted from whole cancer genome sequences, which include complete footprints (including introns and untranslated exons) of protein coding genes, suggesting that the underlying mechanism generating strand bias is restricted to exons (Figures 5 and 7). Examining only the exon compartments of the whole cancer genome sequences reveals the presence of this strand bias in samples with substantial exposure to Signature 2, supporting this conclusion. This result is biologically surprising and the mechanism underlying this difference in strand bias between exons and introns is currently unknown.

DISCUSSION

We have modeled the signatures of somatic mutational processes in cancer genomes as a blind source separation

Figure 3. Evaluating Factors Affecting the Efficacy of Deciphering Mutational Signatures with Simulated Data

(A) Evaluating the effect of deciphering similar mutational signatures from mutational catalogs containing different number of cancer genomes. Signatures III and IV were simulated with cosine similarity between 0.9 and 1.0 (i.e., with extremely similar shapes) whereas the remaining two signatures were very different from any of the other signatures (Figure S1A).

(B) Evaluating the effect of deciphering mutational signatures with different similarities between them from mutational catalogs of 20 cancer genomes.

(C) Evaluating the effect of deciphering different number of mutational signatures from sets of mutational catalogs derived from 10, 20, 30, 50, 70, 100, and 200 cancer genomes.

(D) Evaluating the effect of deciphering different number of mutational signatures from sets of mutational catalogs derived from 50 cancer genomes. The catalogs were simulated with different average number of mutations in a cancer genome.

(E) Evaluating the effect of deciphering two, three, five, or seven mutational signatures from large sets of mutational catalogs containing small number of average mutations per cancer genome. The line colors correspond to the ones in (D) legend.

(F) Evaluating the effect of deciphering mutational signatures with different contributions across sets of 50 mutational catalogs. Signature I's contributions were fixed to contribute a fixed percentage of all mutations in either the whole set of mutational catalogs, i.e., the overall contribution is fixed but different genomes can have different contributions of Signature I (blue bars) or in each individual cancer genome, i.e., Signature I's contributions are fixed in every single mutational catalog (red bars).

(G) Comparison, across all performed simulations, between the accuracy for deciphering mutational signatures and the deciphering error for identifying the contributions of these signatures. The deciphering Frobenius reconstruction error was calculated and averaged for each contribution and normalized based on the number of mutations in the respective mutational catalog. In all panels, deciphering accuracy is shown in cosine similarity where accuracy of 1.00 corresponds to extracting exactly the same process used to simulate the data.

The error bars represent the SD of the deciphering accuracies after performing each simulation scenario 100 times.

See also Figure S1.

Cell PRESS
Open ACCESS

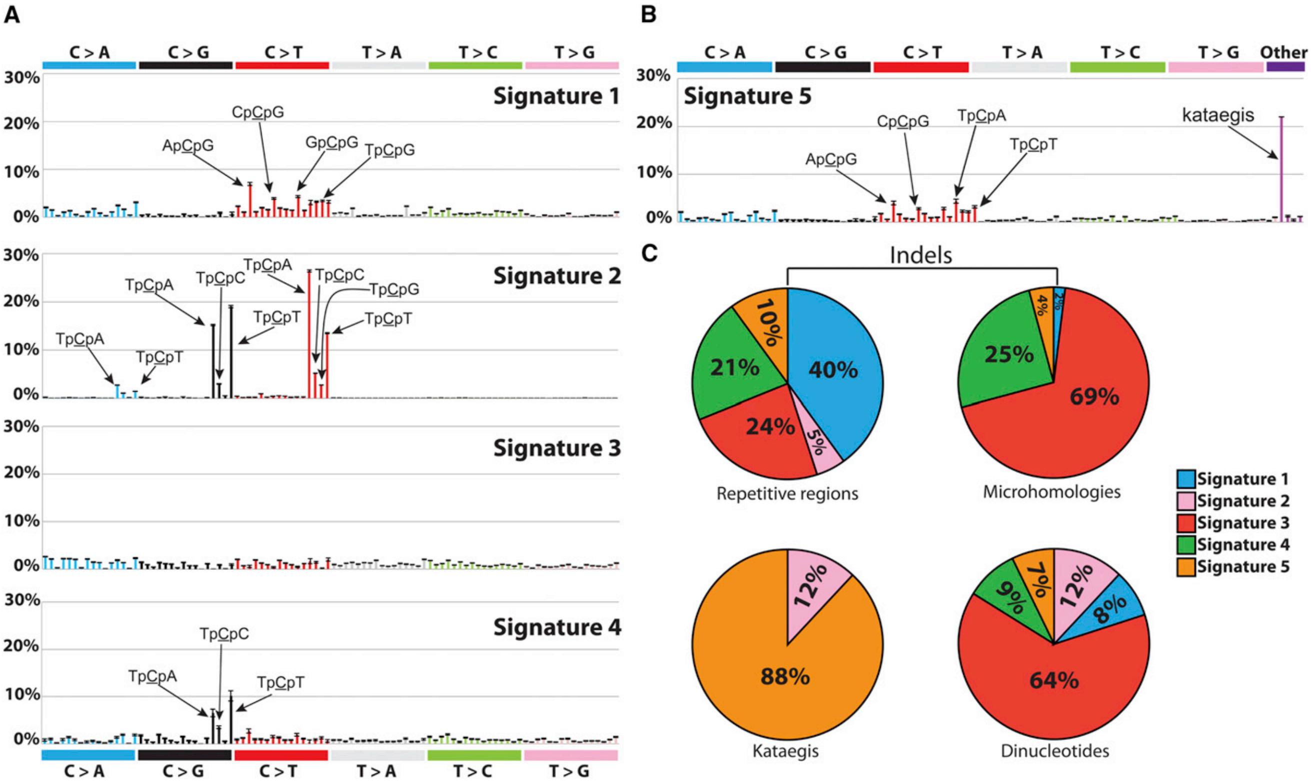

Figure 4. Signatures of Mutational Processes Extracted from the Mutational Catalogs of 21 Breast Cancer Genomes

(A) Four mutational signatures deciphered from the base substitutions (including their immediate 3′ and 5′ sequence context) identified in the 21 breast cancer genomes.

(B) A fifth mutational signature identified when kataegis, dinucleotide substitutions, and indels at microhomologies and at mono or polynucleotide repeats are added as mutation types.

(C) Total contributions of mutations of the five signatures for kataegis, dinucleotide substitutions, and indels in the 21 breast cancer genomes.

The error bars represent the SD of the contributions for each mutation type for the deciphered signature.

See also Figure S2.

problem and introduced a computational framework that extracts these mutational signatures from the mutational catalogs obtained from cancer genome sequences. To identify these signatures, the intrinsic nonnegativity of mutations mandates employment of a method incorporating a nonnegative constraint and our simulations demonstrate that NMF is effective in deciphering mutational signatures from mutational catalogs.

Incorporating additional constraints in NMF could further improve its efficiency. For example, a strong sparsity constraint could be applied to the exposure matrix *E* guaranteeing that the mutational catalog of a cancer genome is described by a minimum number of processes. Algorithms implementing this and other constraints have been previously developed (Berry et al., 2007; Gao and Church, 2005; Peharz and Pernkopf, 2012; Zheng et al., 2006) and could be applied to cancer genomics data. Nevertheless, this study demonstrates that an approach based on the simplest (i.e., without additional constraints) NMF algorithm is sufficient to decipher signatures of mutational processes from catalogs of mutation from cancer genomes.

Parameters to which solutions are sensitive include the number of operative mutational processes, the strength of their exposures, the degree of difference between mutational signatures, the number of analyzed cancer genomes, the number of mutations per cancer genome, and the number of mutation types that are incorporated into the model. These factors will determine the manner in which the method will be applied to future data sets. Importantly, the results show that, despite relatively few mutations present in each case, the approach can be applied to exome data, extracting at least some of the signatures.

Although diverse mutation classes can be included and analyzed by our computational framework, the choice of these classes will largely depend on prior biological knowledge, the available experimental data and perhaps on cancer type. Thus application of our approach can, if desired, be limited to single base substitutions or be widened to include double nucleotide substitutions, indels, geographically localized forms of mutation such as kataegis and mutation features such as transcriptional strand bias. Following this principle, rearrangements and copy number changes (and potentially even epigenetic changes)

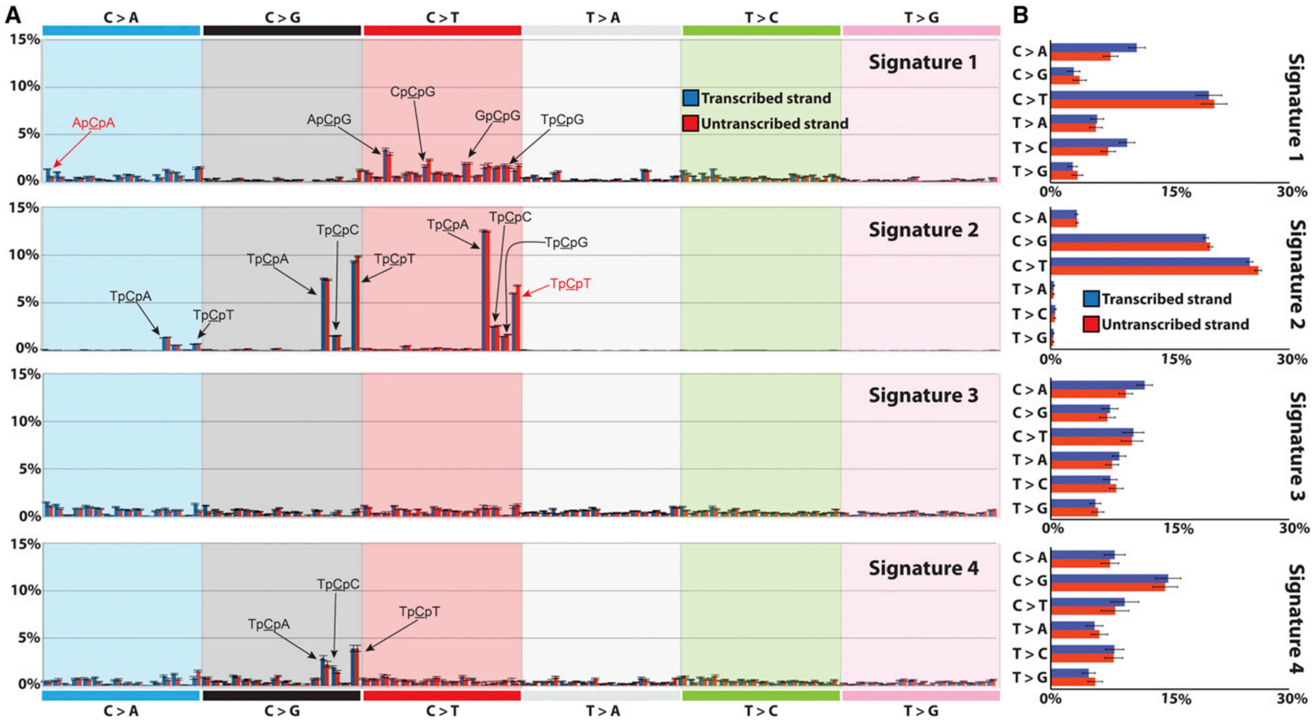

Figure 5. Strand Bias in Signatures of Mutational Processes Extracted from Genic Regions of 21 Breast Cancer Genomes

(A) Four mutational signatures deciphered from the base substitutions (including their immediate 3′ and 5′ sequence context) identified in genic regions of 21 breast cancer genomes.

(B) Sequence context independent summary of strand bias in the four mutational signatures extracted from the 21 breast cancer genomes.

The error bars represent the SD of the contributions for each mutation type for the deciphered signature.

could be incorporated, such that a comprehensive overview of operative mutational processes could be derived. Further, the approach can then be used to estimate the contribution of each mutation process to each cancer and also to time the activity of each process (Nik-Zainal et al., 2012b).

The complexity of the mutational processes operative in some cancers and the inherent challenges in extracting their attendant mutational signatures should not be underestimated. For example, the mutational catalog of a lung cancer in a tobacco smoker will carry the signature of ~60 chemicals that bind and mutate DNA (Pleasance et al., 2010). Each of these chemicals may have its unique mutational signature. A group of smokers loyal to the same brand will be simultaneously exposed to the same combination of mutagens. Analysis of tumors from this group of individuals therefore may not allow the mutagens to be distinguished from one another and our model will extract one signature that encompasses the combined mutational activity of all ~60 chemicals. However, as different cigarette brands may contain different combinations and amounts of mutagens, analysis of mutational catalogs from cancers due to different tobacco brands could allow differentiation between the signatures of each of the different chemicals. An ambitious aspiration of this nature would, however, probably only be feasible with data from thousands of cases, coupled to the statistical power and resolution provided by whole genome mutational catalogs. It should be noted, that even the availability of tens of thousands of cancer genomes may not allow deciphering of the full complexity of all mutational processes occurring in the cancer cells of a person, who has been exposed to various mutagens and treatments throughout his/her lifetime. Nevertheless, our approach allows deciphering the signatures of the most prevalent processes and as the amount of available cancer genomics data increases, it will allow better understanding cellular processes and mutagenesis.

In our first set of experiments using data from breast cancer genomes, we have already extracted mutational signatures for which the underlying biological process is not known. It is highly likely that further cryptic mutational signatures will be extracted once thousands of cancers have been analyzed. Understanding the biological basis of these signatures will be the next imperative. One major approach to achieving this will be to extract mutational signatures from systems (e.g., human cells, mice, yeast, bacteria) with known exposures to mutagens and/or known or engineered changes in DNA editing and repair. Matching of cryptic mutational signatures found in naturally occurring cancers to signatures generated in experimental systems will provide clues to their provenance. These approaches, applied to mutational signatures derived from thousands of human tumors, promise to provide substantial

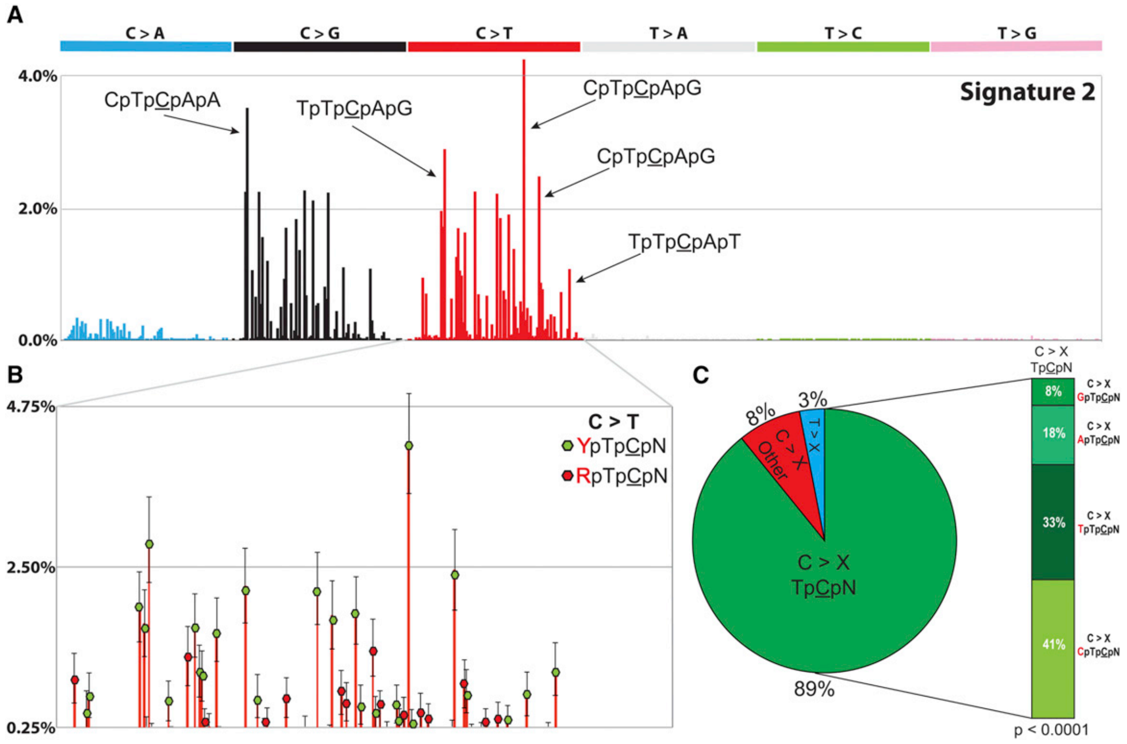

Figure 6. Signatures of Mutational Processes Extended to Include Additional Sequence Context

(A) Signature 2 deciphered from the base substitutions (including the two bases 5′ and 3′ to each mutated base resulting in 1,536 possible mutated pentanucleotides) identified in 21 breast cancer genomes.

(B) Detailed view of C > T mutation types in Signature 2. Purine nucleotides located two bases 5′ of the mutated base are shown in green whereas pyrimidine nucleotides are in red.

(C) Summary of all mutation types caused by Signature 2.

The error bars represent the SD of the contributions for each mutation type for the deciphered signature.

insights into the DNA damage and repair processes that underlie somatic mutagenesis across the spectrum of human cancer.

EXPERIMENTAL PROCEDURES

Model Definition

Mutation type is mathematically represented as a letter from a K-letter alphabet Ξ. Mutational signature is defined as a discrete probability density function over the domain of mutation types in Ξ, $P : \Xi \rightarrow \mathbf{R}_{+}^{K}$. Thus, a signature of a mutational process P_1 can be expressed as a nonnegative K-tuple, $P_1 = [p_1^1, p_1^2, \ldots p_1^K]^T$, where $\sum_{k=1}^{K} p_1^k = 1$ and p_1^k is the probability of the mutational processes P_1 to cause the mutation type corresponding to the k-th letter of the alphabet Ξ. Hence, a set of N mutational signatures can be expressed as a nonnegative mutational signature matrix $P = \begin{bmatrix} p_1^1 & p_2^1 & \cdots & p_{N-1}^1 & p_N^1 \\ \vdots & \vdots & \ddots & \vdots & \vdots \\ p_1^K & p_2^K & \cdots & p_{N-1}^K & p_N^K \end{bmatrix}$ with size $K \times N$, where K is the number of mutation types and N is the number of signatures. The subscript index indicates the signature, whereas the superscript index corresponds to the mutation type.

Exposure to a mutational process P_1 with signature $P_1 = [p_1^1, p_1^2, \ldots p_1^K]^T$ is the number of mutations, $e_g^1 \in \mathbf{N}_0$, attributed to that signature in genome g. In this notation, the product $p_1^2 \times e_g^1$ is the average number of mutations of type corresponding to the second letter of alphabet Ξ caused by the mutational process P_1 in a cancer genome with number g. Hence, we can express the exposure of G genomes to a set of N processes as a nonnegative matrix $E = \begin{bmatrix} e_1^1 & e_2^1 & \cdots & e_{G-1}^1 & e_G^1 \\ \vdots & \vdots & \ddots & \vdots & \vdots \\ e_1^N & e_2^N & \cdots & e_{G-1}^N & e_G^N \end{bmatrix}$ with size $N \times G$. Here, the subscript index indicates the genome whereas the superscript index corresponds to the signature.

The mutational catalog of a cancer genome g defined over the alphabet of mutation types Ξ is represented by $m_g : \Xi \rightarrow \mathbf{N}_0^K$. For a given genome, $g = 1$, its mutational catalog can be expressed as a nonnegative K-tuple, $m_1 = [m_1^1, m_1^2, \ldots m_1^K]^T$. Hence, the mutational catalogs of G cancer genomes can be expressed as a nonnegative mutational catalogs matrix $M = \begin{bmatrix} m_1^1 & m_2^1 & \cdots & m_{G-1}^1 & m_G^1 \\ \vdots & \vdots & \ddots & \vdots & \vdots \\ m_1^K & m_2^K & \cdots & m_{G-1}^K & m_G^K \end{bmatrix}$ of size $K \times G$. In this case, the genomes form the columns of the matrix, where K is the number of mutation types and G is the number of genomes. The subscript index indicates the genome whereas the superscript index corresponds to the mutation type.

In our model, the mutational catalog of a cancer genome is examined as a linear superposition of the signatures of the mutational processes operative in this genome and their respective exposures. This can be expressed for a set of G genomes and N mutational signatures as $M \approx P \times E$. The approximate equality is due to nonsystematic errors and sampling noise.

Framework for Deciphering Signatures of Mutational Processes

For a given set of mutational catalogs M that contain G cancer genomes defined over an alphabet Ξ with K letters corresponding to mutation types

Cell PRESS
Open ACCESS

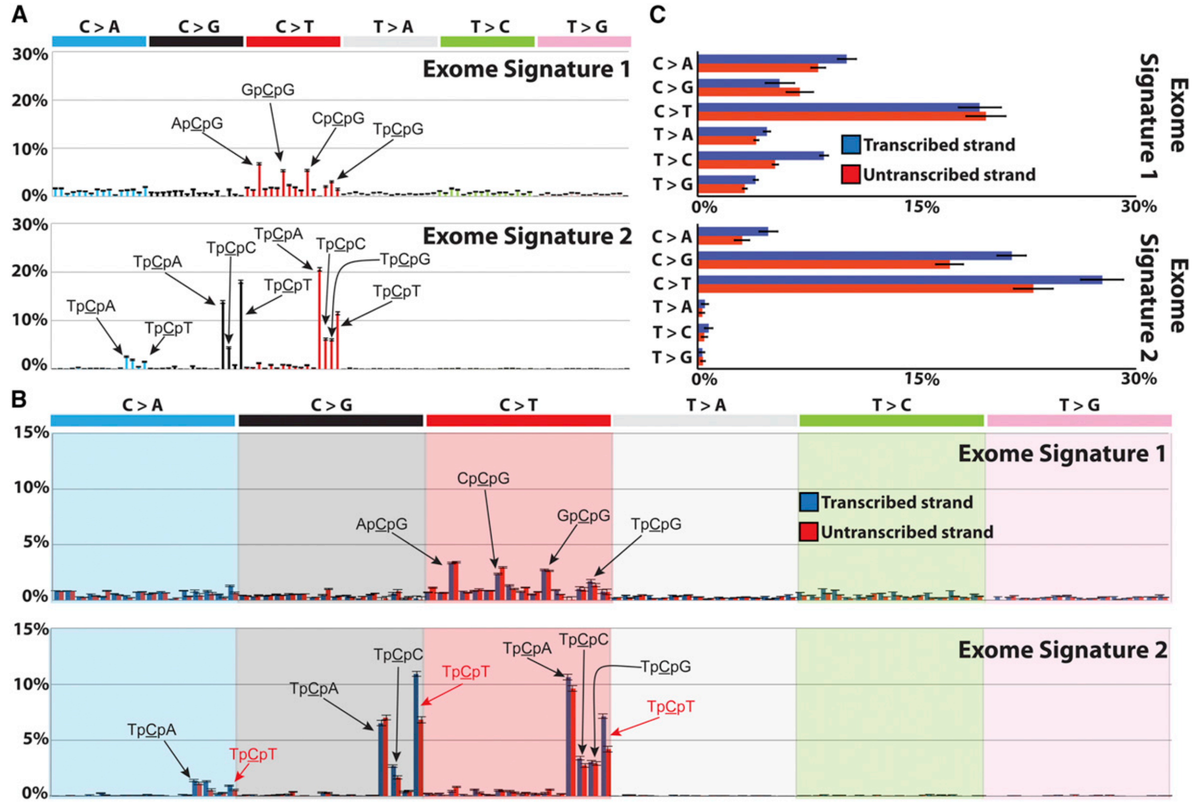

Figure 7. Signatures of Mutational Processes Extracted from the Mutational Catalogs of 100 Breast Cancer Exomes

(A) Two mutational signatures deciphered from the base substitutions (including their immediate 3′ and 5′ sequence context) identified in the exomes of 100 breast cancers.

(B) Strand bias signatures deciphered from the base substitutions identified in the exomes of 100 breast cancers.

(C) Sequence context independent summary of strand bias in the two mutational signatures extracted from the 100 breast cancer exomes.

The error bars represent the SD of the contributions for each mutation type for the deciphered signature.

(i.e., M has a size $K \times G$), we extract N mutational signatures defined over the same alphabet Ξ by applying the algorithm below:

Step 1 (Dimension Reduction)

Reduce the dimensions of the original matrix M by removing any mutation types that together account for $\leq 1\%$ of the mutations in all genomes, i.e., remove the maximum set of rows R in M for which:

$$\sum_{r \in R} \sum_{g=1}^{G} m_g^r \leq 0.01 \times \sum_{k=1}^{K} \sum_{g=1}^{G} m_g^k,$$

and the cardinality of the set R, $|R|$, is maximized. The matrix M is transformed into a new matrix $\dot{M}$ with dimensions $\dot{K} \times G$, where $\dot{K} = K - |R|$.

Step 2 (Bootstrap)

Apply Monte Carlo bootstrap resampling to the dimensionally reduced matrix $\dot{M}$ resulting in a new matrix $\breve{M}$, where the probability for getting a mutation of type corresponding to the qth letter in the alphabet Ξ in a genome g is $\Pr(\breve{m}_g^q) = \dot{m}_g^q / \sum_{k=1}^{K} \dot{m}_g^k$ whereas the total number of mutations in each genome g remains unaffected, i.e., $\sum_{k=1}^{K} \breve{m}_g^k = \sum_{k=1}^{K} \dot{m}_g^k$.

Step 3 (NMF)

Apply the multiplicative update algorithm (Lee and Seung, 1999) for nonnegative matrix factorization to the bootstrapped data by finding the solution to $\min\limits_{P \in \mathbf{M}_{\mathbb{R}_+}^{(\dot{K},N)}, E \in \mathbf{M}_{\mathbb{R}_+}^{(N,G)}} \|\breve{M} - P \times E\|_F^2$:

1. Initialize matrices P and E as random nonnegative matrices with respective sizes $\dot{\mathbf{K}} \times \mathbf{G}$ and $\mathbf{N} \times \mathbf{G}$, where N is the number of signatures.
2. Iterate until convergence, defined as 10,000 iterations without change, or until the maximum number of 1,000,000 iterations is reached:

$$e_G^N \leftarrow e_G^N \frac{[P^T \breve{M}]_{N,G}}{[P^T PE]_{N,G}}$$

$$p_N^{\dot{K}} \leftarrow p_N^{\dot{K}} \frac{[\breve{M} E^T]_{\dot{K},N}}{[PEE^T]_{\dot{K},N}}$$

The notation $[AB]_{x,y}$ is equivalent to the $(x, y)^{th}$ element of the matrix C, where $C = A \times B$.

3. Store the identified signatures P and their respective exposures E.

Although there are many freely available and commercial implementations of the multiplicative update algorithm (Lee and Seung, 1999), the results reported here were deriving mostly using the implementation in Brunet et al. (2004).

Step 4 (Iterate)

Perform Steps 2 and 3 for I iterations. I is determined by evaluating the convergence of the iteration-averaged signature matrix $\overline{P}$ (see below for deriving $\overline{P}$). I is selected in a way such that performing $2 * I$ iterations (i.e., doubling the iterations) does not significantly change $\overline{P}$. In most cases between 400 and 500 iterations are needed, however, sometimes solutions could be found for $I \leq 100$ whereas in rare cases more than 1,000 iterations might be required. In general, the value of I is strongly dependent on the size and type of the initial matrix M.

Step 5 (Cluster)

The iterations performed in Step 4 result in two sets of matrices, $S_P \in \mathbf{M}_{\mathbf{R}_+}^{(\dot{K},N)}$ and $S_E \in \mathbf{M}_{\mathbf{R}_+}^{(\dot{K},N)}$, that correspond respectively to the mutational signatures and their exposures generated over the I iterations. A partition-clustering algorithm was applied to the set of matrices S_P to cluster the data into N clusters. A variation of k-means (Jain, 2010), where each signature for $\forall P \in S_p$ is assigned to exactly one cluster, was used to partition the data. Similarities between mutational signatures were calculated using a cosine similarity (see below) whereas the N centroids were calculated by averaging the signatures belonging to each cluster. The iteration-averaged matrix $\overline{P}$ was formed by combining the N centroid vectors ordered by their reproducibility (see Step 6). The error bars reported for each mutation type in each signature in $\overline{P}$ were calculated as the SD of the corresponding mutation type in each centroid over the I iterations. Note that clustering the data in S_P effectively results in clustering S_E as each signature unambiguously corresponds to exactly one exposure, thus allowing derivation of $\overline{E}$.

Step 6 (Evaluate)

The reproducibility of the derived average signatures $\overline{P}$ is evaluated by examining the tightness and separation of the clusters used to form the centroids in $\overline{P}$ (see Step 5). More specifically, using cosine similarity, the average silhouette width for each of the N clusters is calculated. An average silhouette width of 1.00 is equivalent to consistently deciphering the same mutational signature, whereas a low silhouette width indicates lack of reproducibility of the solution. The average silhouette width (Rousseeuw, 1987) of the N clusters is used as a measure of reproducibility for the whole solution. In addition to reproducibility, the average Frobenius reconstruction error is used to evaluate the accuracy with which the deciphered mutational signatures and their respective exposures describe the original matrix M, i.e., $\|M - \overline{P} \times \overline{E}\|_F^2$, where lower Frobenius reconstruction error corresponds to better describing the original matrix. There is some association between the reproducibility of a solution and its reconstruction error. For example, solutions with very low reproducibility may have iteration inconsistent high Frobenius reconstruction errors. Last, comparison between two mutational signatures A and B, each defined for K mutation types, is done using cosine similarity:

$$\mathrm{sim}(A,B) = \frac{\sum_{k=1}^{K} A_k B_k}{\sqrt{\sum_{k=1}^{K} (A_k)^2}\sqrt{\sum_{k=1}^{K} (B_k)^2}}.$$

Because the elements of A and B are nonnegative, the cosine similarity has a range between 0 and 1. When the cosine similarity is 1 between two signatures, these signatures are exactly the same. In contrast, when the similarity is 0, the signatures are independent.

Model Selection Approach

Our framework for deciphering signatures of mutational processes relies on two input parameters, the original matrix M (size $K \times G$) and the number of mutational signatures N to be deciphered from M. However, in most cases, the value of N is unknown and needs to be determined from M. The model selection framework relies on applying the framework for deciphering signatures of mutational processes for values of N between 1 and min(K,G) $-$ 1. The reproducibility and average Frobenius reconstruction error are evaluated for each N. The value of N is selected when decomposing the matrix M results in highly reproducible mutational signatures and low overall reconstruction error.

Simulating Mutational Catalogs of Cancer Genomes

Signatures of mutational processes with different exposures were randomly generated and used to simulate mutational catalogs of cancer genomes. The simulated mutational catalogs were leveraged to assess the ability of our approach to decipher the mutational signatures with which the data were simulated. In most cases (i.e., unless specified otherwise in the main text), the signatures of mutational processes were stochastically generated with similarities between them similar to those previously observed between signatures of mutational processes derived from the mutational catalogs of breast cancer genomes (Nik-Zainal et al., 2012a). Similarly, unless specified otherwise, the contributions of mutational processes were uniformly distributed across the set of simulated cancer genomes whereas the total number of mutations in each mutational catalog was drawn from a distribution comparable to the distribution of the total substitutions found in many human cancer genomes (Greenman et al., 2007; Nik-Zainal et al., 2012a; Stratton, 2011; Wood et al., 2007). For every mutational process with signature $P_1 = [p_1^1, p_1^2, \ldots p_1^K]^T$ contributing e_g^1 mutations in a cancer genome g, each mutation is assigned to one of the K mutation types according to the discrete probability density function of P_1. Poisson noise was added to every simulated mutational catalog. Lastly, each simulation scenario was repeated 100 times and the SD of the results over these 100 repeats are reported as error bars in the respective figures.

Examined Mutation Types

Mutational catalogs were derived for each of the analyzed samples from the previously identified substitution and indels for the 21 breast cancer whole-genomes (Nik-Zainal et al., 2012a) and 100 breast cancer whole-exomes (Stephens et al., 2012). The immediate 5′ and 3′ sequence context was extracted using the ENSEMBL Core APIs for human genome build GRCh37. Dinucleotide substitutions were identified when two substitutions were present in consecutive bases on the same chromosome (sequence context was ignored). The immediate 5′ and 3′ sequence content of all indels was examined and the ones present at mono/polynucleotide repeats or microhomologies were included in the analyzed mutational catalogs as their respective types. Kataegis substitutions were identified based on their intermutation distances (regardless of sequence context) and excluded from the other substitutions. Strand bias catalogs were derived for each sample using only substitutions identified in the transcribed regions of well-annotated protein coding genes.

Source Code

The framework for deciphering signatures of mutational processes—including its source code, brief documentation, mutational catalogs of the 21 breast cancer whole-genomes, mutational catalogs of 100 breast cancer whole-exomes, and examples (that reproduce results presented in this article) of applying it to these mutational catalogs—are freely available for download from http://www.mathworks.com/matlabcentral/fileexchange/38724.

SUPPLEMENTAL INFORMATION

Supplemental Information includes two figures and can be found with this article online at http://dx.doi.org/10.1016/j.celrep.2012.12.008.

LICENSING INFORMATION

This is an open-access article distributed under the terms of the Creative Commons Attribution License, which permits unrestricted use, distribution, and reproduction in any medium, provided the original author and source are credited.

ACKNOWLEDGMENTS

This work was supported by the Wellcome Trust (grant 098051). S.N.Z. is a Wellcome Trust Clinical Research Training Fellow. P.J.C. is a Wellcome Trust Senior Clinical Research Fellow (grant WT088340MA). We would like to thank Dr. Richard M. Durbin for useful discussions.

Received: August 14, 2012
Revised: November 7, 2012
Accepted: December 13, 2012
Published: January 10, 2013

REFERENCES

Ames, B.N., and Gold, L.S. (1991). Endogenous mutagens and the causes of aging and cancer. Mutat. Res. *250*, 3–16.

Berry, M.W., Browne, M., Langville, A.N., Pauca, V.P., and Plemmons, R.J. (2007). Algorithms and applications for approximate nonnegative matrix factorization. Comput. Stat. Data Anal. *52*, 155–173.

Berwick, M., and Vineis, P. (2000). Markers of DNA repair and susceptibility to cancer in humans: an epidemiologic review. J. Natl. Cancer Inst. *92*, 874–897.

Brunet, J.P., Tamayo, P., Golub, T.R., and Mesirov, J.P. (2004). Metagenes and molecular pattern discovery using matrix factorization. Proc. Natl. Acad. Sci. USA *101*, 4164–4169.

Comon, P. (2010). Handbook of Blind Source Separation: Independent Component Analysis and Blind Deconvolution, First Edition (Boston, MA: Elsevier).

Fuss, J.O., and Cooper, P.K. (2006). DNA repair: dynamic defenders against cancer and aging. PLoS Biol. *4*, e203.

Gao, Y., and Church, G. (2005). Improving molecular cancer class discovery through sparse non-negative matrix factorization. Bioinformatics *21*, 3970–3975.

Greenman, C., Stephens, P., Smith, R., Dalgliesh, G.L., Hunter, C., Bignell, G., Davies, H., Teague, J., Butler, A., Stevens, C., et al. (2007). Patterns of somatic mutation in human cancer genomes. Nature *446*, 153–158.

Hainaut, P., and Pfeifer, G.P. (2001). Patterns of p53 G—>T transversions in lung cancers reflect the primary mutagenic signature of DNA-damage by tobacco smoke. Carcinogenesis *22*, 367–374.

Hudson, T.J., Anderson, W., Artez, A., Barker, A.D., Bell, C., Bernabé, R.R., Bhan, M.K., Calvo, F., Eerola, I., Gerhard, D.S., et al.; International Cancer Genome Consortium. (2010). International network of cancer genome projects. Nature *464*, 993–998.

Hunter, C., Smith, R., Cahill, D.P., Stephens, P., Stevens, C., Teague, J., Greenman, C., Edkins, S., Bignell, G., Davies, H., et al. (2006). A hypermutation phenotype and somatic MSH6 mutations in recurrent human malignant gliomas after alkylator chemotherapy. Cancer Res. *66*, 3987–3991.

Jain, A.K. (2010). Data clustering: 50 years beyond K-means. Pattern Recognit. Lett. *31*, 651–666.

Lee, D.D., and Seung, H.S. (1999). Learning the parts of objects by non-negative matrix factorization. Nature *401*, 788–791.

Nik-Zainal, S., Alexandrov, L.B., Wedge, D.C., Van Loo, P., Greenman, C.D., Raine, K., Jones, D., Hinton, J., Marshall, J., Stebbings, L.A., et al.; Breast Cancer Working Group of the International Cancer Genome Consortium. (2012a). Mutational processes molding the genomes of 21 breast cancers. Cell *149*, 979–993.

Nik-Zainal, S., Van Loo, P., Wedge, D.C., Alexandrov, L.B., Greenman, C.D., Lau, K.W., Raine, K., Jones, D., Marshall, J., Ramakrishna, M., et al.; Breast Cancer Working Group of the International Cancer Genome Consortium. (2012b). The life history of 21 breast cancers. Cell *149*, 994–1007.

Peharz, R., and Pernkopf, F. (2012). Sparse nonnegative matrix factorization with ℓ(0)-constraints. Neurocomputing *80*, 38–46.

Pfeifer, G.P., Denissenko, M.F., Olivier, M., Tretyakova, N., Hecht, S.S., and Hainaut, P. (2002). Tobacco smoke carcinogens, DNA damage and p53 mutations in smoking-associated cancers. Oncogene *21*, 7435–7451.

Pfeifer, G.P., You, Y.H., and Besaratinia, A. (2005). Mutations induced by ultraviolet light. Mutat. Res. *571*, 19–31.

Pleasance, E.D., Stephens, P.J., O'Meara, S., McBride, D.J., Meynert, A., Jones, D., Lin, M.L., Beare, D., Lau, K.W., Greenman, C., et al. (2010). A small-cell lung cancer genome with complex signatures of tobacco exposure. Nature *463*, 184–190.

Pluskota-Karwatka, D. (2008). Modifications of nucleosides by endogenous mutagens-DNA adducts arising from cellular processes. Bioorg. Chem. *36*, 198–213.

Rousseeuw, P.J. (1987). Silhouettes: A graphical aid to the interpretation and validation of cluster analysis. J. Comput. Appl. Math. *20*, 53–65.

Rubin, A.F., and Green, P. (2009). Mutation patterns in cancer genomes. Proc. Natl. Acad. Sci. USA *106*, 21766–21770.

Stephens, P.J., Tarpey, P.S., Davies, H., Van Loo, P., Greenman, C., Wedge, D.C., Zainal, S.N., Martin, S., Varela, I., Bignell, G.R., et al. (2012). The landscape of cancer genes and mutational processes in breast cancer. Nature *486*, 400–404.

Stratton, M.R. (2011). Exploring the genomes of cancer cells: progress and promise. Science *331*, 1553–1558.

Stratton, M.R., Campbell, P.J., and Futreal, P.A. (2009). The cancer genome. Nature *458*, 719–724.

van Zeeland, A.A., Vreeswijk, M.P., de Gruijl, F.R., van Kranen, H.J., Vrieling, H., and Mullenders, L.F. (2005). Transcription-coupled repair: impact on UV-induced mutagenesis in cultured rodent cells and mouse skin tumors. Mutat. Res. *577*, 170–178.

Wood, L.D., Parsons, D.W., Jones, S., Lin, J., Sjöblom, T., Leary, R.J., Shen, D., Boca, S.M., Barber, T., Ptak, J., et al. (2007). The genomic landscapes of human breast and colorectal cancers. Science *318*, 1108–1113.

Zheng, C.-H., Huang, D.-S., Sun, Z.-L., Lyu, M.R., and Lok, T.-M. (2006). Nonnegative independent component analysis based on minimizing mutual information technique. Neurocomputing *69*, 878–883.

Cell PRESS Open ACCESS

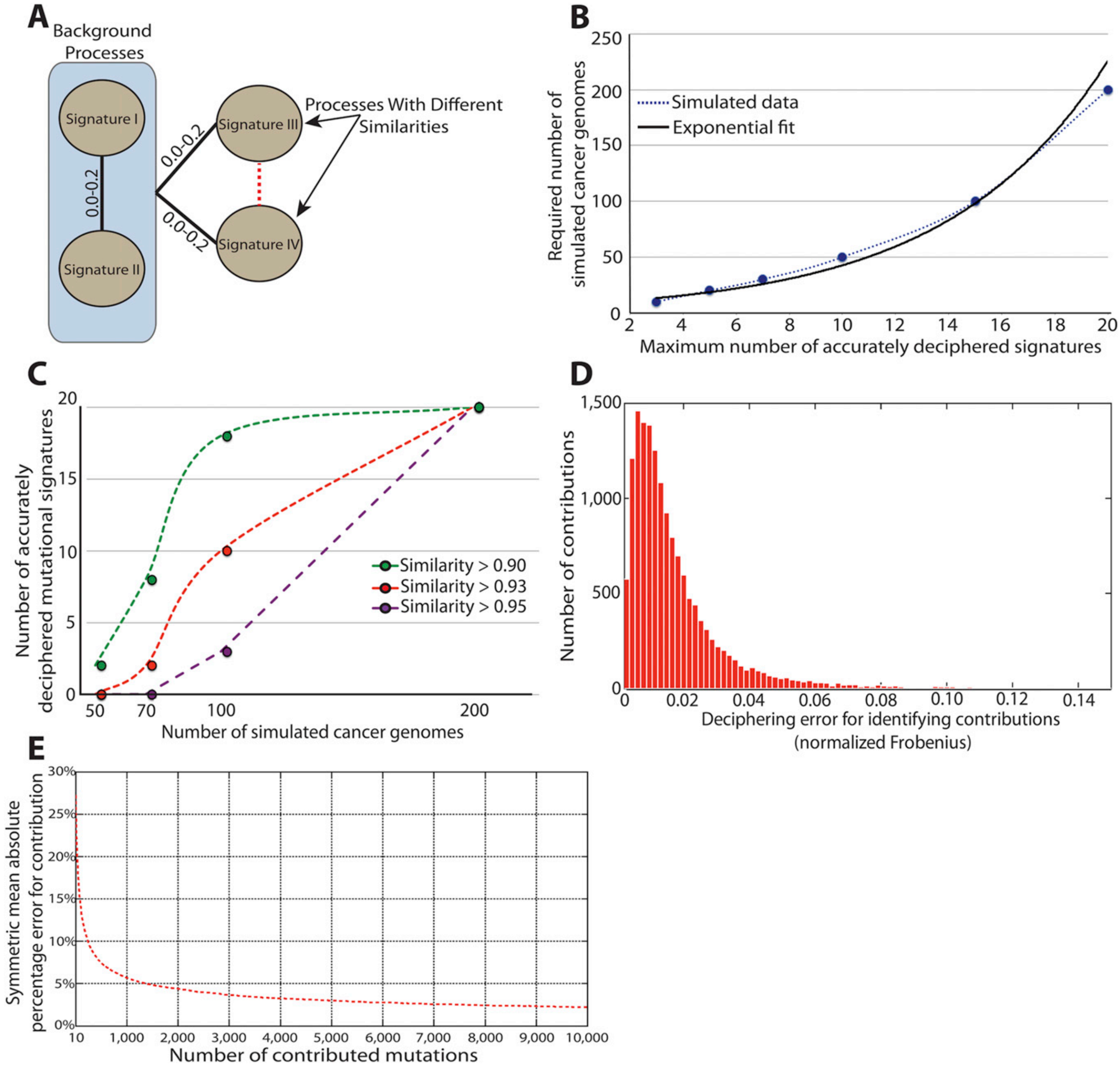

Figure S1. Additional Factors Affecting the Efficacy of Deciphering Mutational Signatures with Simulated Data, Related to Figure 3

(A) Design for simulating the signatures of four mutational processes with different similarities between them. Signatures I and II differ significantly from each other as well as from the other two Signatures (cosine similarity between 0.00 and 0.20). Signatures III and IV were simulated with varying similarities between them.

(B) Dependency between accurately deciphered signatures (i.e., cosine similarity between simulated and deciphered signature > 0.95) and the number of mutational catalogs needed to decipherer these signatures.

(C) Identifying the maximum number of accurately deciphered signatures (cosine similarity between simulated and deciphered signature shown in the legend) from sets of mutational catalogs simulated using the signatures of 20 mutational processes.

(D) Distribution of the normalized Frobenius error for identifying the contributions of accurately deciphered signatures of mutational processes (i.e., cosine similarity between simulated and deciphered signature > 0.95).

(E) Average symmetric mean absolute percentage error for identifying the contributions of accurately deciphered signatures of mutational processes (i.e., cosine similarity between simulated and deciphered signature > 0.95) based on the number mutations contributed by the signature.

Cell PRESS
Open ACCESS

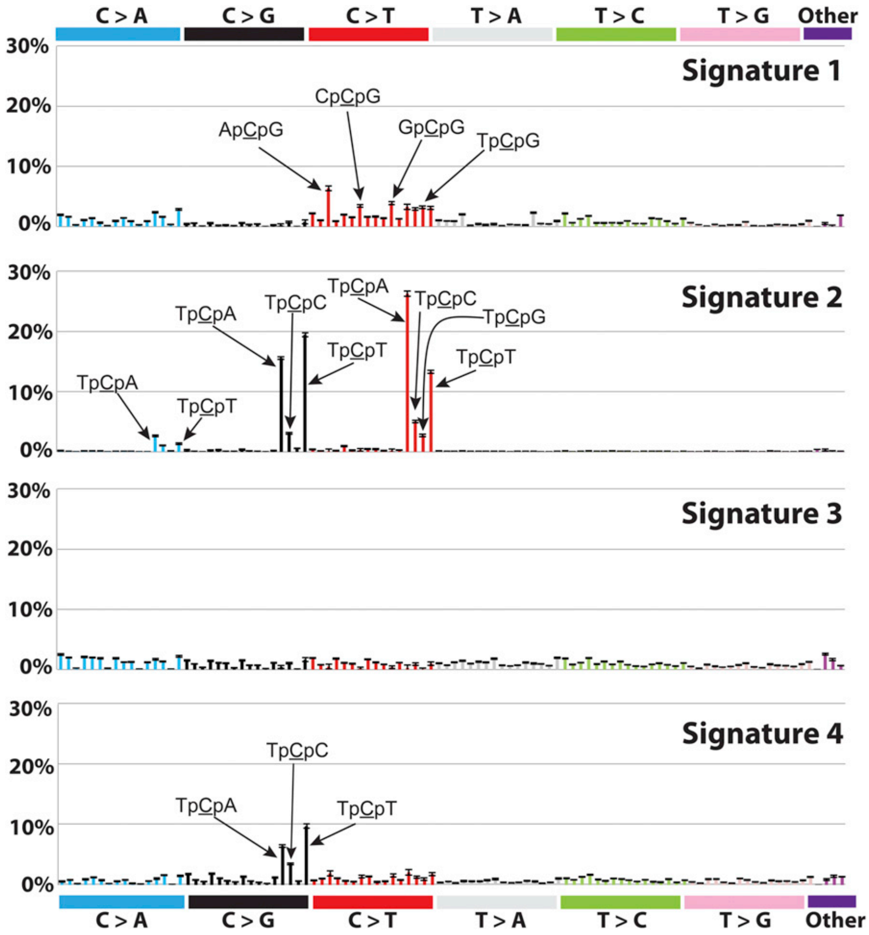

Figure S2. Signatures of Mutational Processes Extracted from the Extended Mutational Catalogs of 21 Breast Cancer Genomes, Related to Figure 4

Four of the five mutational signatures deciphered from the base substitutions (including their immediate 3′ and 5′ sequence context), kataegis, indels, and dinucleotide substitutions identified in the 21 breast cancer genomes. The fifth mutational signature is shown in Figure 4B.
